# Nanochelating based nanocomplex, GFc7, improves quality and quantity of human mesenchymal stem cells during *in vitro* expansion

**DOI:** 10.1186/s13287-015-0216-9

**Published:** 2015-11-23

**Authors:** Maryam Hafizi, Atena Hajarizadeh, Amir Atashi, Somayeh Kalanaky, Saideh Fakharzadeh, Zahra Masoumi, Mohammad Hassan Nazaran, Masoud Soleimani

**Affiliations:** Stem Cell Technology Research Center, Tehran, Iran; Department of Research and Development, Sodour Ahrar Shargh Company, Tehran, Iran; Department of Hematology, Faculty of Medical Sciences, Tarbiat Modares University, Tehran, Iran

**Keywords:** Expansion, GFc7, Human mesenchymal stem cells, Nanocomplex, Nanochelating technology

## Abstract

**Introduction:**

Human mesenchymal stem cells (hMSCs) have been approved for therapeutic applications. Despite the advances in this field, in vitro approaches are still required to improve the essential indices that would pave the way to a bright horizon for an efficient transplantation in the future. Nanotechnology could help to improve these approaches. Studies signified the important role of iron in stem cell metabolism and efficiency of copper chelation application for stem cell expansion

**Methods:**

For the first time, based on novel Nanochelating technology, we design an iron containing copper chelator nano complex, GFc7 and examined on hMSCs during in vitro expansion. In this study, the hMSCs were isolated, characterized and expanded in vitro in two media (with or without GFc7). Then proliferation, cell viability, cell cycle analysis, surface markers, HLADR, pluripotency genes expression, homing and antioxidative defense at genes and protein expression were investigated. Also we analyzed the spontaneous differentiation and examined osteogenic and lipogenic differentiation.

**Results:**

GFc7 affected the expression of key genes, improving both the stemness and fitness of the cells in a precise and balanced manner. We observed significant increases in cell proliferation, enhanced expression of pluripotency genes and homing markers, improved antioxidative defense, repression of genes involved in spontaneous differentiation and exposing the hMSCs to differentiation medium indicated that pretreatment with GFc7 increased the quality and rate of differentiation.

**Conclusions:**

Thus, GFc7 appears to be a potential new supplement for cell culture medium for increasing the efficiency of transplantation.

## Introduction

Human mesenchymal stem cells (hMSCs), a subset of the multipotent non-hematopoietic stem cells found primarily within the bone marrow, have been extensively studied in recent years due to their potential for diverse applications in regenerative medicine [1, 2]. hMSCs have an innate self-renewal capability that is compatible with in vitro expansion systems, enabling maintenance and mass production that is comparable with other stem cells for downstream clinical applications. They can also differentiate into multiple cell types and lineages, including osteoblasts, cardiocytes, and neural cells. In addition, owing to their accessibility and ease of isolation from multiple sources, hMSCs may have a broader application spectrum because they can be readily used for auto-graft transplantation. The HLA-DR-negative nature of hMSCs increases the rate of successful transplantations, and their immunomodulatory features qualify them for treating numerous autoimmune diseases [3–6]. All of these features together have made hMSCs one of the best candidates for a multitude of cell therapy applications but despite the efficient performance of hMSCs and their important advantages, they did not achieve satisfactory effects in some investigations [7, 8] and the question as to what their effective influence is after transplantation remains unanswered [9–11].

The success rate of hMSCs cell therapy significantly decreases due to induced apoptosis in the transplanted cells during the first day of transplantation [8, 12]. It is clear from the literature that this in vitro expansion phase causes dramatic changes in the hMSCs phenotype, which has considerable implications for the development of effective therapies [13].

Recently, to overcome the concomitant problems of the existing procedures, researchers have developed diverse new strategies for in vitro culture. Nanotechnology-based approaches have evolved to provide nanostructures for improving the quantity and fitness of stem cells for more successful in vivo transplantations [14]. Nanochelating technology is a modern field that has the capacity to design and synthetize nano structures via a self-assembly method [15]. In previous studies using this modern technology, Hep-c nano adjuvant and MSc1 nano-complex were synthetized. Hep-c improved cellular immune responses against hepatitis B vaccine [16]. MSc1 nano-complex showed therapeutic behavior in an animal model of multiple sclerosis [17] and neuroprotective effects of three different sizes of nanochelating based nano complexes in MPP(+) induced neurotoxicity [5] were recently reported.

For the first time, based on novel nanochelating technology, we designed and synthesized an iron containing copper chelator nanocomplex (GFc7) via a self-assembly method.

The important role of iron in cell metabolism is clear. Iron is a key cofactor of vital enzymes that are involved in cell cycle promotion [18] and hMSCs proliferation [19], so GFc7 is loaded with iron. On the other hand, several studies reported positive outcomes of using copper chelators for expansion of cord blood hematopoietic stem cells in vitro [20] and in transplantation (phase I/II clinical trial) [19]. Also, an increase in the extracellular concentration of copper modulated the ability of hMSCs to spontaneously differentiate [20]. Based on these reports, GFc7 was designed in a way that has chelating properties with the dominant affinity for copper.

Available reports imply negative effects of oxidative stress on hMSCs functionality [21], expansion and differentiation. Oxidative stress has an important role in the initiation and progression of diseases such as cancer and autoimmune diseases; a huge number of hMSCs recipients have these diseases [22].

Therefore, in this study, we hypothesize that the competent GFc7 structure could increase the efficiency of hMSCs by maximizing antioxidative defense [23] (common cellular model using “H2O2” for evaluating the effect of GFc7), expression of pluripotency markers, cell expansion without spontaneous differentiation, and suitable homing by promoting differentiation potential.

## Materials and methods

### Materials and instrumentation

GFc7 nanocomplex was synthesized by Sodour Ahrar Shargh Company (Tehran, Iran). Minimum essential medium (α-MEM), penicillin G (100 U/ml), streptomycin (100 μg/ml), GlutaMAX, nonessential amino acids, trypsin-EDTA 0.25 %, and phosphate-buffered solution (PBS) were purchased from Gibco (Gibco-Life Technologies, Carlsbad,CA, USA). Hydrogen peroxide (H_2_O_2_), sodium isothiocyanate, dimethyl sulfoxide (DMSO), FeCl_3_, nitric acid, acetone, methanol and formalin, Triton X-100, beta- glycerol phosphate, NHCl, and paraformaldehyde were purchased from Merck (Darmstadt, Germany). AB-human serum, propidium iodide (PI), hydrocortisone, isobutyl methyl xanthine, indomethacin, Oil Red stain, Alizarin Red stain, dexamethasone, ascorbic acid 2-phosphate, 3-(4,5-dimethylthiazol-2-yl)-2,5-diphenyltetrazolium bromide 99 % (MTT), p-nitrophenyl phosphate (pNP), Ficoll, and TRIzol were from Sigma-Aldrich (St Louis, MO, USA). IntraStain kit (Code-Nr.K2311) and all of the antibodies were obtained from Dako (Glostrup, Denmark) and Standard SYBR Green PCR kit from Fermentas, St. Leon-Rot, Germany.

The list of the equipment and instruments used is as follows: FACS Calibur (Becton Dickinson, Cockeysville, MD, USA), absorbance micro plate readers (ELx800™; BioTek, Winooski, VT, USA), Rotor Gene 6000 instrument (Corbett, Sydney, Australia), and scanning electron microscope VEGA-TESCAN-LMU model.

### Experimental overview

Bone marrow collection was approval by Shahid Beheshti University of Medical Sciences, Tehran, Iran (7808) and informed consent was obtained from the patients who participated in the study. The isolation and expansion of hMSCs was performed using the standard protocol [5].

**Fig. 1:**
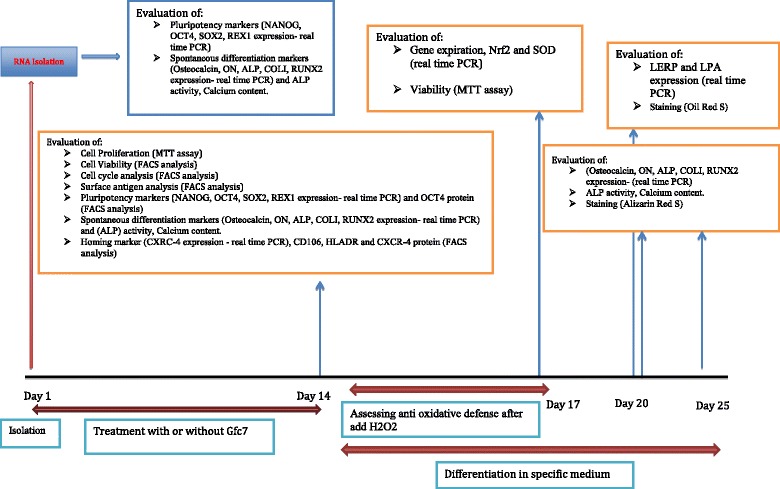
Design and time line of the study

At hMSCs isolation day from bone marrow aspirates, cells were isolated from part of the isolated hMSCs and pluripotency markers and spontaneous differentiation markers were evaluated, Fig. 1.

The remnants of the isolated cells were aliquoted in two parts: control (without GFc7) and test cells (with GFc7); the test cells had GFc7 nanocomplex at a concentration of 0.1 mg/mL in hMSCs specific medium. (This concentration was determined based on seven days treatment of hMSCs with different concentrations of GFc7 and then cell proliferation assay by the MTT method). Then, the following properties were compared in the control and test cells after 14 days culture (*see* Fig. 1):

- Cell viability
- Cell cycle analysis, surface antigen analysis
- Pluripotency markers
- Spontaneous differentiation markers
- Homing marker
- Pluripotency markers
- Spontaneous differentiation markers

After 14 days of incubation, control and test groups were analyzed for differentiation (adipogenic and osteogenic) and antioxidative defense was assessed, Fig. 1.

### Characterization of GFc7 nano-complex

Nanochelating technology [15] was used by the Sodour Ahrar Shargh Company to design and synthesize a novel multi-layered nanosphere, which has an iron donor and copper acceptor structure. This multi-layer nanosphere, synthesized by liquid phase reduction, is called GFc7.

### Synthesis

A) Iron-chelate nanosphere preparation:
  Special sized iron nanospheres were produced based on liquid phase polymerization by using an organic acid. The method does not need protective agents to prevent the agglomeration of the iron-nanospheres.
  Controlling the mole ratio of ferrous sulfate and organic acid can produce special sized iron-nanospheres. First, 1 ml of 0.5 M organic acid was dissolved in 100 ml of H2O with stirring and heating to 90 °C simultaneously. Afterwards, 30 ml of 2.5 mM ferrous sulfate was injected into the solution rapidly and the reaction mixture was maintained at the boiling point for four to seven min before it was allowed to cool to room temperature. When the solution was clear green, the initial iron colloid was condensed by filtering several times to remove unreacted materials to prevent it from agglomerating. The iron-nanospheres can be stable for three days in the dark at 25 °C.
B) Copper-chelator polymerization:
  The prepared iron nanospheres were immersed in 20 mL of saturated glutaric acid solution. After one h, 8 ml ethanol was added; then the solution was heated to 40 °C and stirred slowly for about three h to start growth progression of glutaric acid on the surface of the prepared iron-nanospheres. Afterward, the solution was left to cool for 24 h to precipitate the final GFc7 multi-layer nanospheres. Then, it was filtered and dried at 100 °C.

### Scanning electron microscopy and infrared spectra (IR)

The surface morphology of this nano-complex was characterized using scanning electron microscopy (SEM) at the Razi Metallurgical Research Center.

GFc7 functional groups were characterized by IR in the 400–4,000 cm^−1^ range at the University of Shahid Beheshti.

### Evaluation of GFc7 toxicity

Standard tests were carried out to assess the median lethal dose (LD50) according to the guidelines of the Organization for Economic Co-operation and Development (OECD, guideline 420), in the School of Pharmacy at Tehran University of Medical Sciences [20].

### hMSC isolation and culture

Bone marrow aspirates, collected on ACD-heparin, were used to isolate hMSCs by the Ficoll density gradient protocol. The expansion medium included DMEM F12 supplemented with 10 % human serum, penicillin G, streptomycin, Glutamax and nonessential amino acids. The cells were cultured in flasks and were incubated under a humidified atmosphere with 5 % CO2 at 37 °C.

The cells were then sorted through their surface markers by flow cytometry analysis and their differentiation to osteogenic, adipogenic lineages [5].

### Real-time polymerase chain reaction analysis

Total RNA was extracted using TRIzol according to the manufacturer’s instructions. Synthesis of cDNA was carried out with M-MuLV reverse transcriptase and oligo (dT) primers. Real-time polymerase chain reaction (qRT-PCR) was performed using a standard SYBR Green PCR kit protocol on a Rotor Gene 6000 instrument [24, 25]. Data were normalized to GAPDH as the endogenous control gene. The relative mRNA expression levels were calculated based on the ΔCT method. The list of primers is shown in Table 1.

**Table 1:** Primers for qRT-PCR genes

	Genes	Primer sequences
1	ALP	FW, 5′- GCA CCT GCC TTA CTA ACT C −3′;
		RW, 5′- AGA CAC CCA TCC CAT CTC −3′;
2	COL1	FW, 5′- TGG AGC AAG AGG CGA GAG −3′;
		RW, 5′- CAC CAG CAT CAC CCT TAG C −3′;
3	CXCR4	FW, 5-′ CGC CAC CAA CAG TCA GAG −3′ ;
		RW, 5′- AAC ACA ACC ACC CAC AAG TC −3′;
4	GAPDH	FW, 5′- GACAAGCTTCCCGTTCTCAG-3′;
		RW, 5′- GAGTCAACGGATTTGGTCGT-3′;
5	H-P53	FW, 5′- GGA GTA TTT GGA TGA CAG AAA C −3′;
		RW, 5′- GAT TAC CAC TGG AGT CTT C −3′;
6	LERP	FW, 5′- CAA TCT GAA TGA AAC CAA ACC TC −3′;
		RW, GGC TGC TCC TAT GAT ACC TC −3′;
7	REX1	FW, 5′- CGG GAC GAG GAG TGT TAT TAC −3′;
		RW, 5′- CGT GTT GCT TTG CGA CTT G −3′;
8	RUNX2	FW, 5′- GCC TTC AAG GTG GTA GCC C −3′;
		RW, 5′- CGT TAC CCG CCA TGA CAG TA −3′
9	Lipoprotein lipase	FW, 5′- CCC TAC AAA GTC TTC CAT TAC −3′;
		RW, 5′- AGT TCT CCA ATA TCT ACC TCT G −3′;
10	NRF2	FW, 5′- GCG ACG GAA AGA GTA TGA G −3′;
		RW, 5′- GGG CAA CCT GGG AGT AG −3′;
11	NANOG	FW, 5′- GCT AAG GAC AAC ATT GAT AGA AG −3′;
		RW, 5′- CTT CAT CAC CAA TTC GTA CTT G −3′;
12	Osteonectin	FW: 5′: CTCGCTTCGGCAGCACACATATAC-3′,
		RW: 5′- ACGCTTCACGAATTTGCGTGTC-3′.
13	Osteocalcin	FW, 5-′ GCA AAG GTG CAG CCT TTG TG −3′ ;
		RW, 5′- GGC TCC CAG CCA TTG ATA CAG −3′;
14	OCT-4	FW, 5′- CGC CGT ATG AGT TCT GTG −3′;
		RW, 5′- GGT GAT CCT CTT CTG CTT C −3′;
15	SOX2	FW; 5′- GGA CTG AGA GAA AGA AGA GGA G −3′
		RW, 5′- GAA AAT CAG GCG AAG AAT AAT-3′;
16	SOD1	FW, 5′- CGA GCA GAA GGA AAG TAA TG −3′;
		RW, 5′- TGG ATA GAG GAT TAA AGT GAG G −3′;

### Differentiation potential

After hMSCs maintenance in media with and without GFc7 for 14 days, cells were cultured in specific differentiation (adipogenic and osteogenic) media.

Adipogenic differentiation was confirmed by Oil Red staining analysis. For osteogenic differentiation for indicating calcium mineralization, samples were stained by Alizarin Red. Evaluation of ALP activity and calcium content measurements were performed for osteogenic differentiation. All tests were done according to standard protocols [5, 19].

### Flow cytometry analysis

In all of the following tests, cell surface, intracellular proteins and cell viability assessment were performed by flow cytometry analysis (FACS). Data were analyzed using Win MDI 2.8 software, and the results were illustrated using histograms and dot plots [25].

A) Cell surface:
  A total of 1 × 10^5^ cells was allocated into 2 ml microtubes with 100 μl PBS. Then, they were stained at 4 °C for 30 min with monoclonal antibodies against the following: fluorescein isothiocyanate conjugated human CD34, CD44, CD90, and CD105 and PE-conjugated CD73, CD166, and HLADR. In each test, the suitable isotype matching the antibody was used as control to cover nonspecific binding. Cells were fixed with 1 % paraformaldehyde in PBS.
B) Intracellular proteins:
  For monoclonal antibodies against human OCT4, sample preparation and staining procedures were performed using the IntraStain kit (Code-Nr.K2311) according to the manufacturer’s protocol [21].
C) Cell viability assessment
  To adjust flow cytometer settings for PI, we added staining solution with gentle mixing and incubation for one min in the dark. PI fluorescence was determined with a FACS [25].

### MTT assay

The fallowing tests, were performed by MTT assay according to the standard protocols [17].A) The MTT Cell Proliferation Assay measures the cell proliferation rate.B) Measuring cell-protection capacity against H2O2-induced oxidative injuryIn the experimental studies, H2O2 is used for simulating OS-induced cell death [23]. The hMSCs were plated at a density of 1x104 cells/well in 96-well plates in 100 μl specific medium. The cells were treated with GFc7 (0.01 mg/mL) for 14 days and were then incubated with 5 μM of H2O2 for 72 hours. For the control group, hMSCs cells were cultured without treatment with GFc7 or H2O2 (as negative control) and were treated simply with H2O2 for 72 hours (as positive control), after that, MTT assay and qRT-PCR analysis were performed.

### Measuring cell-protection capacity against H2O2-induced oxidative injury

In the experimental studies, H2O2 is used for simulating OS-induced cell death [23]. The hMSCs were plated at a density of 1x10^4^ cells/well in 96-well plates in 100 μl specific medium. The cells were treated with GFc7 (0.01 mg/mL) for 14 days and were then incubated with 5 μM of H2O2 for 72 hours. For the control group, hMSCs cells were cultured without treatment with GFc7 or H_2_O_2_ (as negative control) and were treated simply with H_2_O_2_ for 72 hours (as positive control), after the MTT assay and qRT-PCR analysis were performed.

### Statistical analysis

Student’s t-test was used to determine whether there was a significant difference between the groups. In order to compare multiple groups, the analysis of variance (ANOVA) test was applied; the difference was considered significant if the P value was lower than 0.05. All data are shown as mean ± standard deviation (SD).

## Results

### GFc7 characterization

**Fig. 2:**
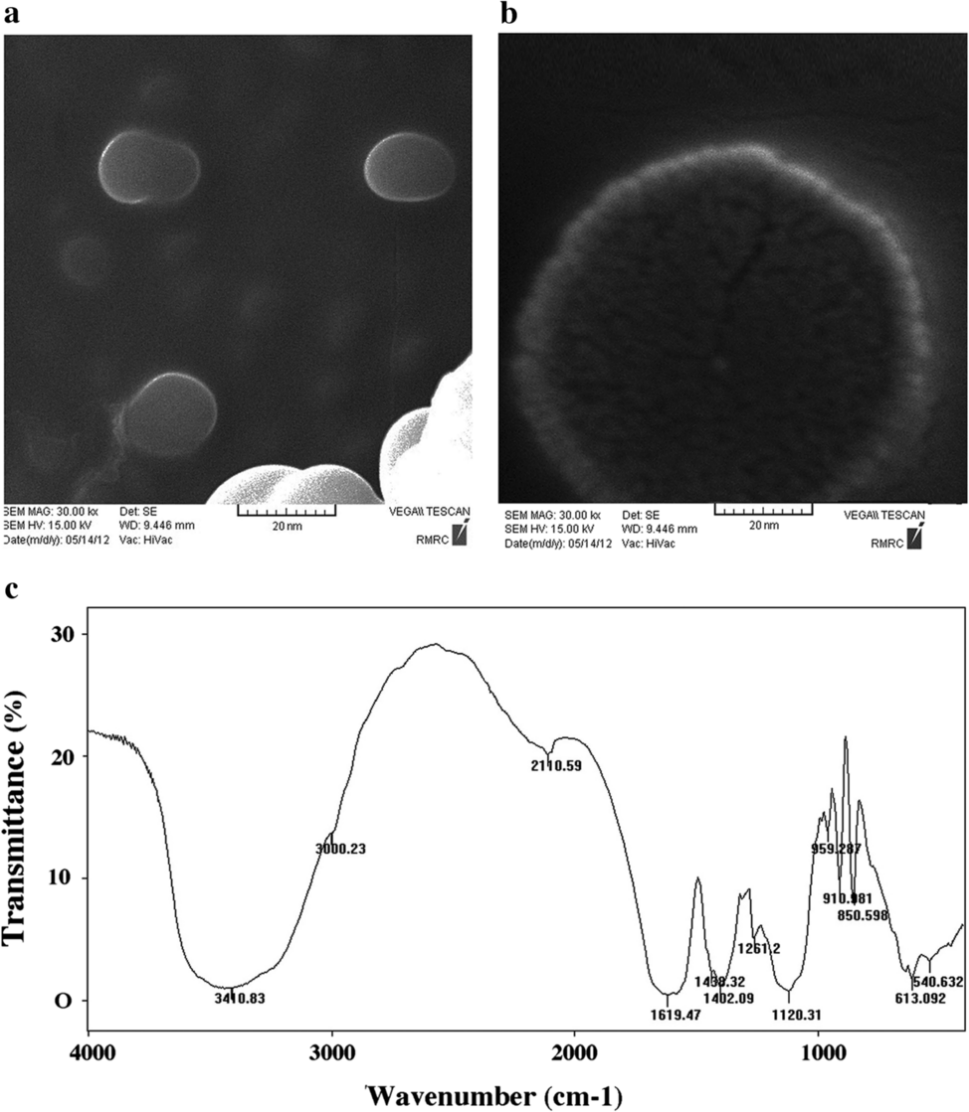
**a** SEM image of Fe-chelate nanosphere (the faces of the Fe-chelate nanospheres are smooth). **b** SEM image of the growth progression of Cu-chelator polymerized on the surface of Fe-chelate nanosphere, bar = 20 nm; **c** IR spectrum. *SEM* scanning electron microscopy, *IR* infrared

SEM images showed that the size of the GFc7 nano-complex in the first synthesis step (iron-chelate nanosphere) was 18 nm (Fig. 2a) and after the progression of the copper-chelator polymerized on the surface of the iron-chelate nanosphere, it was approximately 85 nm (Fig. 2b).

The IR spectrum analyses clearly demonstrated that GFc7 was an organic structure containing carbonyl, alcohol, and amide groups. At 3,400 cm^−1^, carboxylic acid C-H bonds could be found. At 3,000 cm^−1^, O-H stretch is shown. The carboxylic acid structure appears as a wide, broad peak spectrum between 2,000 to 3,000 cm^−1^ (Fig. 2c).

Toxicity reports indicated that the intraperitoneal LD50 of this nano complex was 68.190 mg/kg for mouse and was thus considered to have low toxicity. However, its oral LD50 for mouse was 752.63 mg/kg, classifying it as non-toxic.

### hMSC characterization

**Fig. 3:**
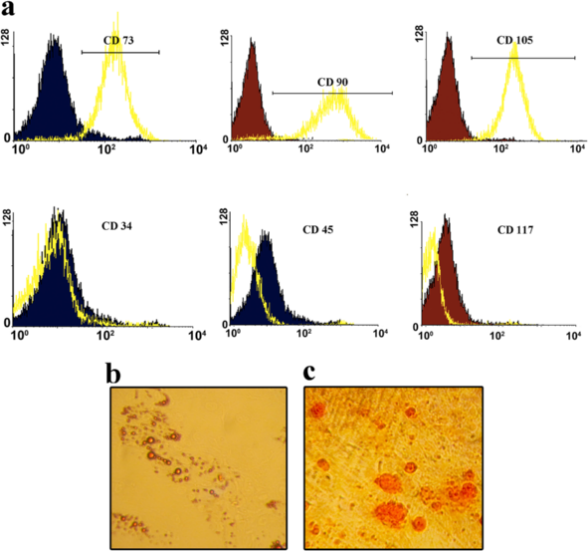
**a** Cells expressed hMSc markers. The cells were negative for CD34, CD45, and CD 117 but were positive for CD 73, CD90, and CD105. **b** The ability for lipogenic differentiation was assessed by staining with Oil Red. **c** The ability for osteogenesis differentiation was assessed by staining with Alizarin (400 X magnification). *hMSc* mesenchymal stem cell.

After isolation and adherence to cell culture flasks, hMSCs were morphologically spindle shaped. The identity of the cells was determined by confirming that the surface markers were positive for CD 90, 73, and 105 and negative for CD 45, 34, and 117 (Fig. 3a) and their differentiation to adipogenic lineages (Fig. 3b) and osteogenic lineages (Fig. 3c).

### GFc7 increased proliferation with a normal cell cycle

**Fig. 4:**
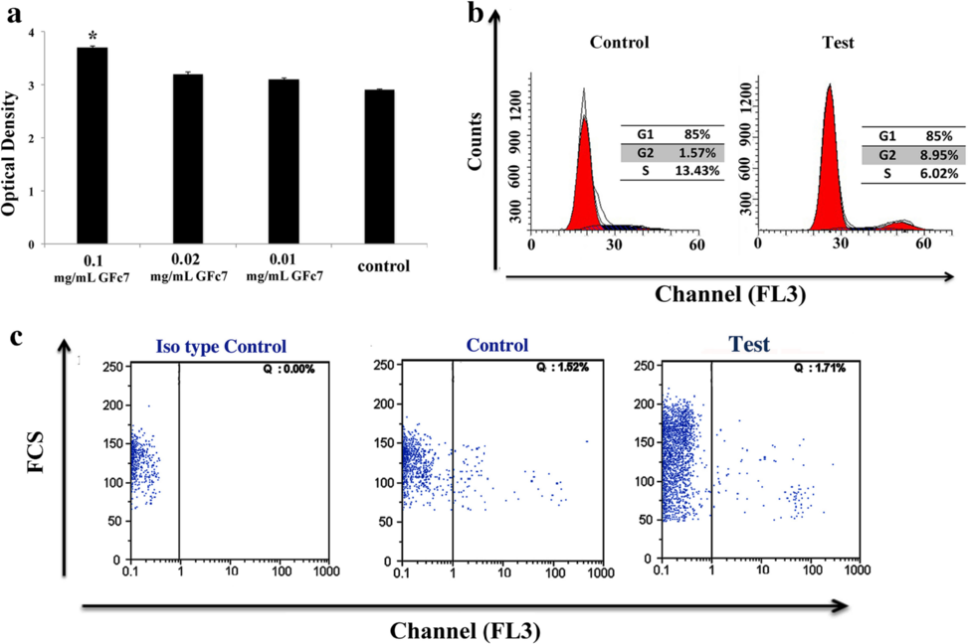
**a** The MTT Cell Proliferation Assay. **b** FACS analysis of cell cycle parameters. **c** Cell viability assays by flow cytometry. (data shown with dot plot diagram). (Isotype control is an antibody of the same isotype as a primary antibody with no relevant specificity to the target antigen and it used as negative controls to help differentiate non-specific background signal from specific antibody signal). Data are expressed as mean ± SD. Asterisks show significant differences with *p* < 0.05. *hMSCs* human mesenchymal stem cells, *PI* propidium iodide

The cells were treated with 0.1 mg/ml, 0.02 mg/ml, and 0.01 mg/ml doses of GFc7 for seven days and proliferation improved, respectively, by 3.7 ± 0.035, 3.1 ± 0.025, and 3.2 ± 0.032-fold and by 2.9 ± 0.026-fold in the control group (Fig. 4a).

The results from the cell cycle analyses in the control and test groups were S = 13.5, G2 = 1.5, G1 = 85 and S = 6, G2 = 9, G1 = 85, respectively. In the control cells, however, decreases in the G2 phase of the cell cycle analysis were noticed (Fig. 4b).

### GFc7 had no negative effects on cell viability

The percentage of cell viability between the test and control groups was equal to 98.5 ± 1 % (Fig. 4c).

### GFc7 efficiently increased hMSCs pluripotency

**Fig. 5:**
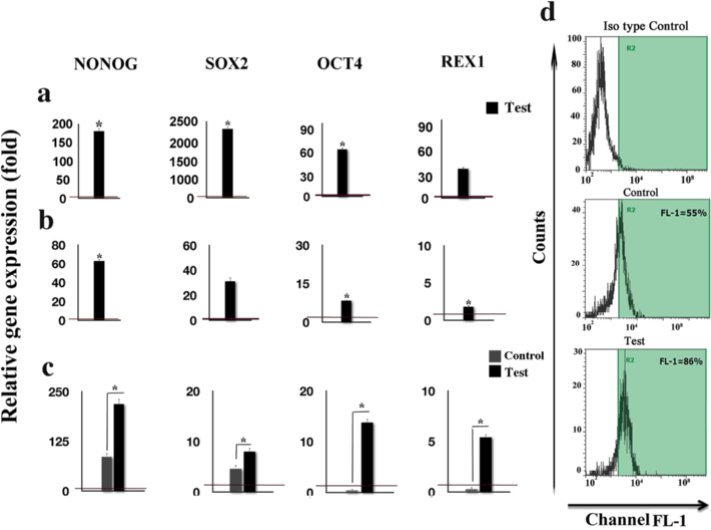
**a** Relative expression level of pluripotency genes (NONOG, SOX2, OCT-4, and REX1) on day 7 of the test compared with control. **b** Relative expression level of pluripotency genes (NONOG, SOX2, OCT-4, and REX1) on day 14 of the test compared with control. **c** Relative expression levels of pluripotency genes (NONOG, SOX2, OCT-4, and REX1) in the two groups (test and control) were compared with the cells obtained on the isolation day. **d** Flow cytometric analysis of Oct-4 expression. The red horizontal line shows the one-fold enrichment cut off criteria. Data are expressed as mean ± SD; Asterisks show significant differences with *p* < 0.05

On day 7, the expression of NANOG, SOX2, OCT4, and REX1 in the test hMSCs increased by 197 ± 10, 2,300 ± 97, 61 ± 8 and 30 ± 2-fold, respectively, compared with that of the control cells (Fig. 5a).

On day 14, the expression of NANOG, SOX2, OCT4, and REX1 in the test cells increased respectively by 63 ± 4, 31 ± 7, 8.3 ± 1, and 1.8 ± 0.05-fold compared with the control group (Fig. 5b).

Comparing these two groups with the cells from the isolation day verified maintenance of pluripotency in the treated cells. In the test cells, the expression of NANOG, SOX2, OCT4, and REX1 increased by 218 ± 30, 8± 0.4,14 ± 1 and 6  ± 1-fold, while in the control cells, the expression increased by 85.4 ± 10,4.6±0.3, 0.4 ± 0.03 and 0.3 ± 0.01-fold, respectively. (Fig. 5c).

The histogram graphs demonstrated that OCT4 expression increased by 30 % in the test cells relative to the control (Fig. 5d).

### GFc7 inhibited spontaneous differentiation

**Fig. 6:**
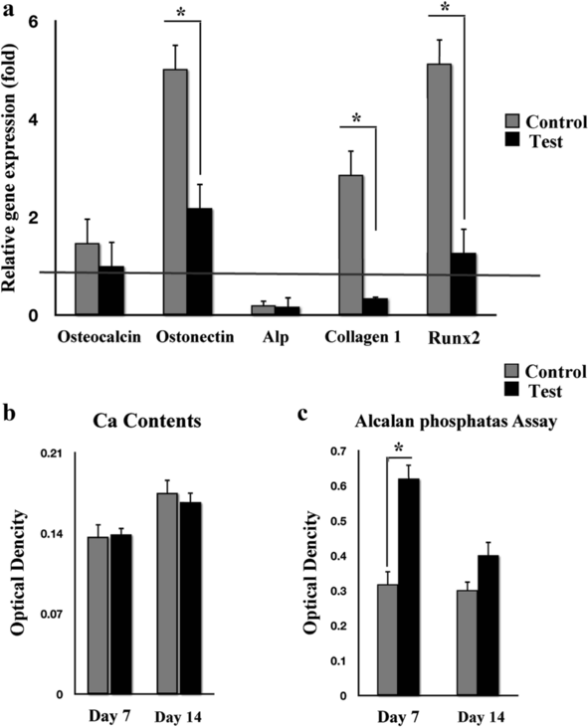
**a** Effect of GFc7 nanocomplex on spontaneous differentiation, relative expression of osteogenic differentiation genes (OCN, ON, ALP, COLI, and RUNX2) in control and test groups were compared to the cells obtained on the isolation day. **b** Effect of GFc7 on spontaneous differentiation on ALP activity of hMSCs in the control and test groups compared to the cells obtained on the isolation day. **c** Effect of GFc7 on spontaneous differentiation on calcium content of hMSCs in the control and test groups compared to the cells obtained on the isolation day. The red horizontal line shows the one-fold enrichment cut off criteria. Data are expressed as mean ± SD. Asterisks show significant differences with *p* < 0.05. *OCN* osteocalcin, *ON* osteonectin, *ALP* alkaline phosphatase

The main issue in cell therapy is the lack of spontaneous differentiation of the cells during in vitro culture. In this study, the cells were examined for the expression of osteogenic specific genes. Relative to the cells obtained on isolation day, the expression of osteocalcin (OCN), osteonectin (ON), alkaline phosphatase (ALP), COLI, and RUNX2 in the test group changed by 1 ± 0.9, 2.2 ± 0.5, 0.17 ± 0.8, 0.34 ± 0.04, and 1.27 ± 1.2-fold, respectively. In the control group, these values increased by 1.5 ± 1.4, 5.02 ± 1, 0.2 ± 0.1, 2.94 ± 1.5, and 5.1 ± 1-fold, respective to the cells from the isolation day (Fig. 6a). Although calcium content was equal (Fig. 6b), GFc7 induced greater levels of ALP in the test cells compared to the control group (Fig. 6c).

### GFc7 promoted hMSCs-specific markers

After 14 days of cell proliferation, the expression levels of surface antigens and the mean fluorescence intensity (MFI) of MoAb-reacted proteins [26] were analyzed in different groups (control and test). Data are shown in the Tables 2 and 3.

**Table 2:** Percent of CD markers between groups

CD marker	Control	Test
CD73	70 %	91 %
CD90	93 %	96 %
CD105	92 %	99.5 %
CD 106	Negative	19 %
CD34	Negative	Negative
CD44	Negative	Negative
CD166	Negative	Negative
HLADR	Negative	2 %

**Table 3:** Percent of mean fluorescence intensity

GMean	Control	Test
CD73	16 %	27 %
CD90	37 %	22 %
CD105	26 %	23 %
CD 106	Negative	11 %
CD34	Negative	Negative
CD44	Negative	Negative
CD166	Negative	Negative
HLADR	Negative	Negative

### Protected hMSCs against H2O2 induced cell toxicity

**Fig. 7:**
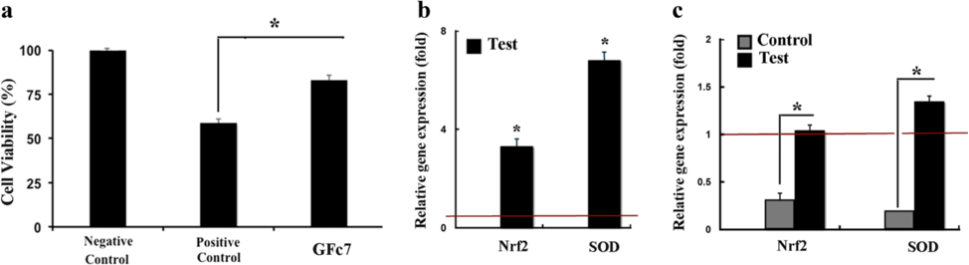
**a** hMSCs protection from H2O2-induced oxidative toxicity by GFc7nono complex. **b** Relative expression of antioxidant gene (Nrf2, SOD) in test group compared to the control after exposure to H2O2. **c** Relative expression of antioxidant gene (Nrf2, SOD) in test and control groups after exposure to H2O2 compared to the cells obtained on the isolation day. The red horizontal line shows the one-fold enrichment cutoff criteria. Data are expressed as mean ± SD. Asterisks show significant differences with *p* < 0.05. *Nrf2* nuclear factor (erythroid-derived 2)-like 2, *SOD* superoxide dismutases

In the positive control group, cell viability was 58.7 ± 2 %. In the test group, cell viability increased significantly to 83 ± 3 %. (Fig. 7a).

Real-time PCR was used to assess nuclear factor (erythroid-derived 2)-like 2 (Nrf2) and superoxide dismutases (SOD) gene expression at the optimum dose, demonstrating a 3.3 ± 0.5-fold increase (Nrf2) and a 6.6 ± 0.8-fold increase (SOD) in the test group relative to the controls (Fig. 7b). Nrf2 and SOD gene expression was 1.04 ± 0.02 and 1.3 ± 0.03 in the test group and 0.314 ± 0.02 and 0.198 ± 0.01in the control group relative to the isolation test (Fig. 7c).

### GFc7 efficiently increased markers associated with cell homing

**Fig. 8:**
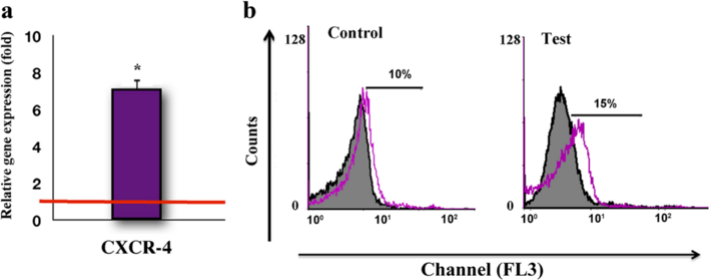
**a** Relative expression of CXCR-4 for test compared with control. **b** Flow cytometric analysis of CXCR-4 expression protein. The red horizontal line shows the one-fold enrichment cutoff criteria. Data are expressed as mean ± SD. Asterisks show significant differences with *p* < 0.05. The dark filled histogram represents the isotype control; the color line histogram represents expression of CXCR4 on hMSCs. *CXCR-4* C-X-C chemokine receptor type 4

After 14 days of cell proliferation, under the optimal dose of GFc7, C-X-C chemokine receptor type 4 (CXCR-4) expression increased by 8 ± 1-fold (Fig. 8a), and its protein level increased by 5 ± 0.5 % in comparison with the control group (Fig. 8b). hMSCs are known to be negative for HLADR, but as observed in the flow cytometry graph, the quantity of HLADR in the control cells was 2 %, compared with 0 % in the test groups. This result demonstrated that GFc7 preserved the suppression of HLADR expression during treatment (Table 2). Unlike the expression of CD106 in the control group (0 %), the expression of CD106 in the treated cells was approximately 20 ± 1 % (Table 2).

### GFc7 promoted quality and rate of hMSCs differentiation

After pretreating hMSCs with the GFc7, the cells were put through adipogenic differentiation in specific medium. A higher rate of differentiation in the pretreated cells, compared against the control, was confirmed by various tests.

**Fig. 9:**
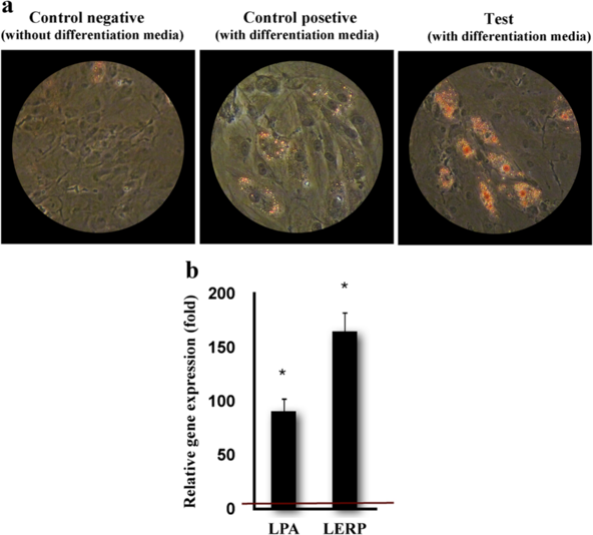
**a** The ability for adipogenesis differentiation was assessed by staining with Oil Red O for lipid accumulation (400 X magnification). **b** Relative expression of lipogenic-specific genes (LERP, LPA) in test compared with control. The red horizontal line shows the one-fold enrichment cutoff criteria. Data are expressed as mean ± SD. Asterisks show significant differences with *p* < 0.05. *LPA* lysophosphatidic acid

The percentage of adipogenic differentiation capability of hMSCs by Oil Red staining after five days of induction between the test and control groups was 75 ± 6 % and 25 ± 4 %, respectively (Fig. 9a) and expression of the adipogenic genes LERP and LPA on day 5 improved, respectively, by 164 ± 20 and 90 ± 20.1-fold compared with the control (Fig. 9b).

**Fig. 10:**
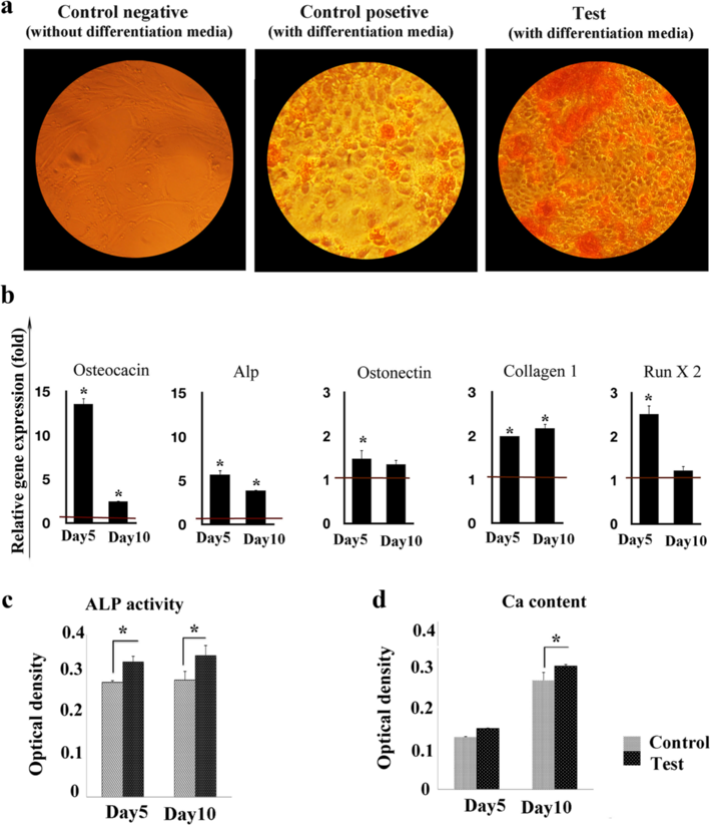
**a** The ability for osteogenesis differentiation was assessed by staining with Alizarin Red for calcium deposition (400 X magnification). **b** Relative expression of osteogenic-specific genes (OCN, ON, ALP, COLI, and RUNX2) for test group on days 7 and 14 compared with control. **c** ALP activity of hMSCs in the control and test groups during osteogenic differentiation (day 5 and 10). **d** Calcium content of hMSCs in the control and test groups during osteogenic differentiation (day 5 and 10). The red horizontal line shows the one-fold enrichment cut off criteria. Data are expressed as mean ± SD. Asterisks show significant differences with *p* < 0.05

In the osteogenic differentiation, nodules of mineral aggregations could be observed which were stained by Alizarin Red S. Staining was carried out on day 10 of the differentiation process and indicated a higher rate of differentiation in the treated cells compared to the control. The percentage of differentiation among the test and control cells was 50 ± 5 % and 20 ± 4 %, respectively (Fig. 10a).

Compared with the control in the osteogenic differentiation assay the expression of OCN, ON, ALP, COLI, and RUNX2 in test cells grew, respectively, by 13.5 ± 2, 1.5 ± 0.2, 5.7 ± 1, 2 ± 0, and 2.5 ± 0.5-fold at the fifth day after the initiation of the differentiation process and by 0.9 ± 0.01, 0.3 ± 0.01, 1 ± 0.02, 0.1 ± 0.02 and 0.6 ± 0.1-fold ten days after initiating the differentiation process (Fig. 10b). Calcium content assays indicated that the Ca^2+^ level in the control and test cells was 0.62 ± 0.01 and 0.96 ± 0.132, respectively, after five days of osteogenic treatment and 2.7 ± 0.48 and 3.5 ± 0.51 after ten days of differentiation (Fig. 10c). ALP activity level was measured and found to be 0.142 ± 0.005 (control) and 0.163 ± 0.01 (test) after five days and 0.32 ± 0.01 and 0.34 ± 0.01 after ten days (Fig. 10d).

## Discussion

Human mesenchymal stem cells are undifferentiated cells responsible for the growth, homeostasis and repair of many tissues [27]. Although stem cells hold great potential for the treatment of many injuries and degenerative diseases, several obstacles must be overcome before their therapeutic application can be realized [28]. The application of nanotechnology to stem cell biology would be able to address these challenges [29].

Nanostructures are of particular interest because they have the advantageous features of a high surface-to-volume ratio and small size (1–100 nm) [30] and they elicit a higher degree of biological plasticity compared with conventional micro- or macro-structures [31]. In the field of biomaterial development and in vivo implant technology, the nanoscale structure and morphologic factor of the surface have played a critical role in accelerating the rate of cell proliferation and enhancing tissue acceptance with a reduced immune response [31].

Particle size is the most important and most studied factor affecting nanoparticle toxicity. Nanoparticles that are too small (e.g., <10 nm), cause damage. In contrast, particles >100 nm do not possess the desired pharmacological properties for effective delivery [32].

Recently nanochelating technology [15] synthetized and introduced novel nano complexes based on a self- assembly method [16, 17, 33].

In the present study, Gfc7 as a novel iron containing copper chelator efficiently and in a balanced manner increased hMSCs quantity and quality characters. SEM images showed that first we had a Fe-chelate nanosphere, then growth progression of Cu-chelator polymerized on the surface of the Fe-chelate nanosphere.

Also the SEM image showed that the size of the GFc7 nano-complex was 85 nm and the IR spectrum analyses demonstrated that this nanocomplex was an organic structure.

Assessing the influence of GFc7 on the pluripotency potential of hMSCs indicated that GFc7 could significantly increase cell proliferation and simultaneously improve the expression of the pluripotency markers OCT4, SOX2, NANOG, and REX1 at day 7 and 14 after isolation compared with control cells.

The physiological role of iron in cell proliferation is a known concept, because several important enzymes in the cell cycle progression and DNA synthesis are iron dependent [34]. Huang et al. evaluated the effects of superparamagnetic iron oxide nanoparticles (SPIONs) on hMSCs proliferation and reported that Ferucarbotran, an ionic SPIO, increases hMSCs cell numbers and growth [35].

On the other hand, several studies have shown the role of copper in stem cell differentiation [20]. Peled et al. studied the effects of copper-chloride, copper chelator tetraethylenepentamine (TEPA) and TEPA/copper mixtures on short- and long-term cord blood-derived CD34 (+) cell cultures. The results showed that addition of TEPA, TEPA/copper mixtures up to equimolar concentrations, and the TEPA-Cu complex to CD34(+) cultures resulted in inhibition of differentiation and enhancement of long-term self-renewal of CD34(+) cells which was correlated with reduction in the cellular chelatable Cu content [36]. Also in the study by Rodríguez et al., copper supplementation diminished the proliferation rate of MSCs, increasing their ability to differentiate into the osteogenic and the adipogenic lineages [37].

It is notable that the expression of these pluripotency markers in GFc7 treated cells was higher compared to the isolation day. In the study by Choudhery et al., they reported that advancing age negatively impacts stem cell function and such age related alterations may be detrimental for successful stem cell therapies. They showed that aged hMSCs displayed senescent features, reduced viability and proliferation and also differentiation potential when compared with cells isolated from young donors [38]. So the increase of pluripotency markers in GFc7 treated cells, compared to the isolation day, suggests the potential of this nano complex as an option for functional improvement of older patients’ MSCs.

One of the major concerns regarding the in vitro culture of hMSCs includes their spontaneous differentiation into lineages such as osteogenic cells [39]. Notably, treatment with GFc7 led to a stable level of RUNX-2, an early marker that triggers the osteogenic differentiation cascade [40], and other osteogenic genes in comparison with the cells obtained on isolation day. However, the expression of RUNX2, ON, and COLI exhibited dramatic increases in the control cells, which signified the beginning of unsuitable differentiation events at genomic levels. Conversely, the GFc7 nano-complex induced greater levels of ALP activity. Considering that ALP is a stemness marker for hMSCs derived from bone marrow, embryonic stem cells, and other sources, GFc7 treatment could be a way to maintain stemness capacity in the test cells [41].

Nrf2 is a transcription factor known as the master regulator of the antioxidative stress response, which protects the cells by upgrading antioxidative defenses [42]. Several studies have demonstrated that Nrf2 is up-regulated by hydrogen peroxide, hypoxia, and serum deprivation stresses, and this is in favor of the cell viability [43, 44]. Nrf2 acts in part by enhancing SODs, key enzymes that reduce reactive superoxide forms [45]. GFc7 could efficiently protect hMSCs viability against H2O2 induced oxidative stress and induce expression of Nrf2 and SOD compared to control cells. These findings suggest that the nano complex sufficiently promoted the antioxidative defense by improving the expression of these transcription factors against oxidative stress.

Adhesion molecules such as CD106 are among the most fundamental factors in improving the migration rate of transplanted cells from the injection site into the damaged tissues [46] Furthermore, stem cells with intrinsic expression of CXCR-4 on their surfaces have been shown to be suitable candidates in cell therapy and regenerative medicine [47]. Considering the vital roles of CD106 (adhesion molecules) [48] and CXCR-4 in successful cell transplantation, the improved expression of homing markers along with the lack of HLADR expression [49] under GFc7 treatment could promise an improvement in the transplantation of GFc7-treated cells to the damaged areas [46].

The improvement of differentiation potential toward osteogenic or adipogenic features in special differentiation media was confirmed by various tests. It is notable that fat vacuoles (in adipogenic cells) were clearly observed in the cytoplasm of pretreated hMSCs merely five days after adipogenic differentiation, while no sign of differentiation was noticed among the controls in the same time period. Importantly, observation of fat vacuoles mostly happened after seven days of transferring in specific differentiation medium [50]. The expression of LPA and LERP was also assessed, demonstrating significant enhancements in the treated cells, compared to the control.

The enhancement of osteogenic-specific genes was also noticed in the treated cells five days after exposure to osteogenic differentiation medium. It is noteworthy that the expression of RUNX2 was followed by the promotion of COLI at early stages of differentiation and ALP and OCN at later stages [51]. This enhancement seemed to be functional, resulting in significantly higher ALP activity and calcium content in the test cells compared with the control. CD106^+^ hMSCs have proven to be more capable of differentiating into osteogenic lineages [51, 52]. The increase in the expression of this protein in the treated cells was further evidence of osteogenic differentiation promotion among these cells.

Altogether, it could be perceived that along with the increased quality of the treated cells, the duration of differentiation was decreased. This was a perfectly purposeful improvement, as the pluripotency characteristics of the cells were efficiently preserved in the medium containing GFc7, and the treated cells differentiated faster and were more competent after exposure to differentiation medium [50].

## Conclusions

The results of our study, including:: nano scale size 10 nm < e.g., < 100 nm of GFc7; self-assembly synthesis method; novel nanocomplex (an iron containing copper chelator nano complex); low toxicity in vivo; concomitant enhancement of four pluripotency genes; inhibition of spontaneous differentiation; improved cell proliferation along with maintaining and increasing the pluripotency characteristics of hMSCs; an appropriate increase in Nrf2 during exposure to OS; protection of hMSCs against OS-induced damages; and promotion of the quality of hMSCs differentiation by accelerating the differentiation process and increasing the expression of osteogenic- or adipogenic-associated genes.

According to our results, GFc7 with a novel structure has simultaneously affected the apex of the cellular regulatory system and the lower sections.

In conclusion, this study showed that the GFc7 nano-complex has the potential to be evaluated in complementary studies as an efficient supplement for hMSCs expansion.

## Abbreviations

ALP: Alkaline phosphatase
CXCR-4: C-X-C chemokine receptor type 4
DMSO: Sodium isothiocyanate, dimethyl sulfoxide
GFc7: Iron containing copper chelator nano complex
H_2_O_2_: Hydrogen peroxide
hMSCs: Human mesenchymal stem cells
IR: Infrared spectra
LPA: Lysophosphatidic acid
MFI: Mean fluorescence intensity
MTT: 3-(4,5-dimethylthiazol-2-yl)-2,5-diphenyltetrazolium bromide 99 %
Nrf2: Nuclear factor (erythroid-derived 2)-like 2
OCN: Osteocalcin
ON: Osteonectin
PBS: Phosphate-buffered solution
PI: Propidium iodide
pNP: p-nitrophenyl phosphate
qRT-PCR: Real-time polymerase chain reaction
SEM: Scanning electron microscopy
SOD: Superoxide dismutases
SPIONs: Superparamagnetic iron oxide nanoparticles
TEPA: Copper chelator tetraethylenepentamine
α-MEM: Minimum essential medium
